# A multi-center, prospective, open-label, 8-week study of certoparin for anticoagulation during maintenance hemodialysis – the membrane study

**DOI:** 10.1186/1471-2369-13-50

**Published:** 2012-06-28

**Authors:** Oliver Dorsch, Detlef H Krieter, Horst-Dieter Lemke, Stefan Fischer, Nima Melzer, Christian Sieder, Peter Bramlage, Job Harenberg

**Affiliations:** 1 KfH Kuratorium für Dialyse und Nierentransplantation e.V., KfH Nierenzentrum, Friesener Straße 37a, 96317, Kronach, Germany; 2 Universitätsklinik Würzburg, Nephrologie, Würzburg, Germany; 3 EXcorLab GmbH, Obernburg, Germany; 4 Dialyse Centrum Darmstadt, Darmstadt, Germany; 5 Novartis Pharma GmbH, Nürnberg, Germany; 6 Institut für Pharmakologie und präventive Medizin, Mahlow, Germany; 7 Klinische Pharmakologie Mannheim, Ruprecht-Karls-Universität Heidelberg, Mannheim, Germany

## Abstract

**Background:**

Adequate anticoagulation is prerequisite for effective hemodialysis to prevent clotting in the extracorporeal circuit. We aimed providing first data on the efficacy and safety of the low-molecular-weight heparin certoparin in this setting.

**Methods:**

Multicenter, open-label, 8-week trial. Patients received a single dose of 3,000 IU certoparin i.v. with additional titration steps of 600 IU and/or continuous infusion if necessary.

**Results:**

120 patients were screened, 109 enrolled (median age 71; range 26–90 years) and 106 available for efficacy analyses. The percentage of unsatisfactory dialysis results at 8 weeks due to clotting or bleeding, was 1.9% (n = 2/106; 95% confidence interval [CI] 0.23–6.65%); no major bleeding. 1.9% had moderate/severe clotting in the lines/bubble catcher and 2.8% in the dialyser at week 8. 15.7 ± 14.3% of the dialysis filters’ visual surface area was showing redness. In subgroups of patients receiving median doses of 3000 ± 0, 3000 (2400–6000) and 4200 (3000–6600) IU, plasma aXa levels at baseline, 4 and 8 weeks were 0.24 [95%CI 0.21–0.27], 0.33 [0.27–0.40] and 0.38 [0.33–0.45] aXa IU/ml at 2 h. C_48h_ was 0.01 [0.01–0.02] aXa IU at all visits. At baseline and 4 weeks AUC_0-48h_ was 2.66 [2.19–3.24] and 3.66 [3.00–4.45] aXa IU*h/ml. In 3.0% of dialyses (n = 83/2724) prolonged fistula compression times were documented. Eight patients (7.34%) had at least one episode of minor bleeding. 4) 85.3% of patients had any adverse event, 9.2% were serious without suspected drug relation; and in 32 patients a drug-relation was suspected.

**Conclusions:**

Certoparin appears effective and safe for anticoagulation in patients undergoing maintenance hemodialysis.

## Background

Adequate anticoagulation is a precondition for effective hemodialysis to prevent clotting in the extracorporeal circuit and to improve biocompatibility of artificial membranes [1]. For this purpose, unfractionated heparin (UFH) is currently the most widely used anticoagulant [1,2]. However, low molecular weight heparins (LMWH) have the advantage over UFH in that they show less nonspecific binding to endothelium, macrophages, platelets and plasma proteins, a more predictable anticoagulant response, and usually have a low requirement for monitoring [3]. Furthermore, chronic UFH use may cause a number of untoward effects, such as heparin induced dyslipidemia [4,5], an increased risk of bleeding [6,7], allergic reactions [8], thrombocytopenia [9], osteoporosis [10] and aldosterone suppression [11].

Certoparin was shown to be safe and effective in patients with chronic renal insufficiency in a recent subgroup analysis of the CERTIFY study [12]. Its action may be partially reversed by protamine hydrochloride [13]. Compared to other LMWHs certoparin has an intermediate renal clearance of antiXa activity (3.6-4.1%) [14,15] and body weight independent dosing [16]. The present feasibility trial was designed to provide data on the efficacy and safety of certoparin in the prophylaxis of clotting in the extracorporeal circuit in patients receiving routine hemodialysis 2-3 times per week.

## Methods

MEMBRANE was a multicenter, open-label, non-controlled prospective in patients undergoing maintenance hemodialysis (ClinicalTrials.gov Identifier: NCT01179620). The study was conducted between July 2010 (first patient in) and March 2011 (last patient out) in accordance with the ethical principles of the Declaration of Helsinki. It was reviewed by the Independent Ethics Committee or Institutional Review Board for each participating centre. These included the General Medical Council (Landesärztekammer) in Munich, Frankfurt, Dresden, Bad Segeberg and Münster and the ethic committees of the Technical University Munich, the University of Münster and the University of Magdeburg. Informed consent was obtained from each subject in writing.

The protocol was amended once after the inclusion of 12 patients which introduced the allowance for an infusion of certoparin in case the chosen bolus was not sufficient, added a second pharmacokinetic analysis, and increased the sample size to adjust for the additional inclusion of patients with hemofiltration (HF) and hemodiafiltration (HDF) (for details see the respective paragraphs).

### Principal study design

Studies investigating the efficacy and safety of LMWH for anticoagulation during chronic hemodialysis have used a variety of designs [17] and there is no guidance from the European Medicines Agency (EMA) for setting up such a study. In the 17 randomized, controlled trials which were included in the systematic review of Lim et al. [17], 10 used a randomized open cross-over design, one a blinded cross-over design, and 6 were open trials using a parallel design. Studies ranged in size and duration from 8 to 149 patients and 1 session up to 36 months, respectively. In none of the trials using a parallel control arm, a predefined comparative statistical hypothesis was tested.

Outcomes compared in the systematic review by Lim [17] were bleedings as a parameter determining the safety of the interventions (assessed in 12 studies as bleeding symptoms or access compression times), thrombosis of the extracorporeal circuit as a parameter determining efficacy (assessed in 17 studies) and anti-Xa levels to establish pharmacokinetics (measured in 14 studies). Extracorporeal thrombosis was assessed by visual inspection or scoring of the extent of clotting in the filter system, the lines and bubble catcher or the dialyzer which in all cases was based on the subjective decision of the investigators. One study rated the dialysis result as satisfactory or unsatisfactory according to the scores of the line/bubble catcher and the dialyzer.

During the design of the study we considered a controlled setting with prospective hypothesis testing. For this purpose a placebo-controlled design was not considered adequate because prophylaxis of extracorporeal clotting is widely considered necessary and a standard procedure. For a UFH-controlled design we expected no superiority of certoparin over UFH based on the analysis of Lim [17]. On the other hand a non-inferiority design was not deemed feasible because neither blinding and bias-free assessment nor sensitivity and validation were appropriately achievable.

Therefore it appeared reasonable to aim at determining the proportion of patients with complications during dialysis defined as the presence of clotting or major bleeding using a straightforward, non-controlled design. The assumption was that between 0 and 5% of patients would experience adverse effects at week 8 and patient numbers to verify this were adjusted to comply with this objective. To show this clinical endpoint, no randomization or comparator arm was needed.

### Objectives

The primary objective of MEMBRANE was to investigate the efficacy and safety of certoparin to prevent clotting in the extracorporeal circuit during hemodialysis at week 8. Therefore, the primary endpoint was defined as the percentage of unsatisfactory dialysis results at week 8 due to clotting or bleeding. The dialysis was considered unsatisfactory if a score of 2 (moderate clotting) or 3 (severe clotting) was given to the lines/bubble catcher (vs 0 for no clots and 1 for minimal clots) and/or the dialyser (moderate redness overall or several ≥ 3) small or few large (≥ 2 mm) dark red fibre bundles OR total clotting of the dialyser defined as stop in hemodialysis requiring change of the extracorporeal circuit) or if any clotting required premature interruption of the hemodialysis session (Table 1). Bleeding was considered a reason for unsatisfactory dialysis when leading to premature interruption of the hemodialysis session or in case of major bleeding.

**Table 1:** Criteria for the evaluation of clinical efficacy of clotting prevention as evaluated visually by the investigator at the study site at the end of the hemodialysis after the first session and after week 8

**Score**		**Lines and bubble catcher**	**Dialyser**
Satisfactory	0	No clots	Good, clear dialyser
	1	Minimal clots	Light redness overall or only a few (< 3) and small (< 2 mm) dark red fibre bundles
Unstatisfactory	2	Moderate clotting	Moderate redness overall or several (≥ 3) small or few large (≥ 2 mm) dark red fibre bundles
	3	Severe clotting	Total clotting of the dialyser (stop in hemodialysis, requiring change of the extracorporeal circuit).

The secondary objectives were: 1) To assess the pharmacokinetics of certoparin in subjects receiving regular hemodialysis treatments including hemodiafiltration and hemofiltration, indirectly determined by the concentration-time profile of antifactor Xa activity using a chromogenic assay; 2) to document the safety and tolerability of certoparin over 8 weeks in subjects receiving regular hemodialysis treatments; 3) to assess clinical efficacy by inspection of the filter system (lines, bubble catcher, dialyzer) at the site of dialysis by the staff in the dialysis center; 4) To assess efficacy via the percentage of clotted area in the transversal (= horizontal) plane of the filter (at central lab). Safety endpoints were 1) antiXa activity at the beginning and at the end of the hemodialysis session at week 4 and week 8; 2) clinical and laboratory factors that predict an excessive (anti-) coagulant effect (anti-Xa, D-Dimer); and 3) bleeding events. Adverse events were adjudicated by the local physician responsible for the conduct of the study.

Further to these objectives cases of prolonged shunt compression times were recorded. Normal shunt compression times were 5 to 13 minutes in prior studies as suggested by the meta-analysis by Lim [17]. The degree of “prolongation” was not predefined however because of the high clinical variability and left up to the treating physician. In the amendment exploratory objectives were added to assess the course of dose adaption and the outcome of dialysis over time.

### Patients

The patient population consisted of clinically stable ambulatory patients of at least 18 years with established chronic kidney disease (CKD) stage 5 D requiring chronic hemodialysis (2-3 times per week) of at least 4 h duration per session for at least 3 months. Hemoglobin had to be ≥ 10.0 g/dL at the first visit. It is allowed to administer aspirin (repetitive dosing ≤ 325 mg daily, single dosing of < 1000 mg), ticlopidine, clopidogrel, dipyridamole or their combination. Selected exclusion criteria were: 1) History of clinically significant bleeding within the last 4 weeks; 2) Any acute or chronic illness interfering with coagulation; 3) History of heparin induced thrombocytopenia type II (HIT II); 4) Target blood flow during dialysis of less than 200 ml/min; 5) any concomitant medication with dextran 40, chronic systemic glucocorticoids (≥ 4 months), thrombolytic agents and anticoagulants (e.g. phenprocoumon) or glycoprotein IIb/IIIa antagonists; 6) Acute or history of non-hemorrhagic stroke (< 3 months); hemorrhagic stroke or intracranial bleeding (< 12 months) or stroke for which thrombolytic therapy was planned. After the amendment the physicians were allowed to also include patients with hemofiltration (HF) and hemodialfiltration (HDF).

### Use and dosing of certoparin

Patients, before being connected for dialysis, received a single bolus dose of 3,000 IU certoparin sodium i.v. (Mono-Embolex®; Novartis Pharma GmbH, Germany) via the needle or the catheter but not into the arterial line at 2-3 routinely scheduled hemodialysis sessions per week for a total of 8 weeks. Titration was allowed in dose steps of 600 IU up to a maximum dose of 6,000 IU. These dose adjustments were made at the discretion of the treating physician but not based on routine anti-factor Xa determinations. The dialyser and blood lines were not pre-rinsed with certoparin but only saline before dialysis.

Because of the fast elimination of certoparin documented in the pharmacokinetic analysis A (PK-A, see below), the titration algorithm was changed with the amendment, allowing to increase the bolus dose to a maximum of 4,200 IU. If a satisfactory result was not achieved, patients received a bolus of 3,000 IU plus an infusion of 600 IU/h up to 1 h before the end of the dialysis session. This would result in a total dose of 4,800 IU for patients who were dialyzed for 4 h. If a satisfactory result was still not achieved the bolus was increased stepwise (3,600 IU and 4,200 IU) combined with an infusion of 600 IU/h resulting in cumulative doses of up to 6,600 IU. After completion of dose adaption, the individually titrated certoparin dose was to be used in subsequent dialyses.

### Definition of bleeding complications

Bleeding complications were assessed by the local physician in charge for the study. *Major bleeding* was defined as fatal bleeding, clinically overt bleeding associated with a fall of the haemoglobin concentration greater than 20 g/l compared to baseline, clinically overt bleeding that requires transfusion of two or more units of packed red cells or whole blood, symptomatic bleeding in a critical area or organ, such as intracranial, intraspinal, retroperitoneal, pericardial bleeding. *Minor* bleeding was defined as bleeding events which do not meet the above mentioned criteria. *Arteriovenous fistula bleeding* was recorded when compression time at the end of the dialysis session was prolonged and was recorded separately.

### Pharmacodynamic assessment

In a subgroup of 12 patients in 3 selected centers (referred to as PK-A) blood samples were collected from fistula prior to the dialysis according to a predetermined schedule at the first dialysis session, and at a session after 4 weeks (visit V3). This was done immediately before and up to 48 h after the administration of certoparin with the following schedule: immediately before certoparin administration (0 min), at 1 minute, 30 minutes, 1 h, 2 h, 3 h, 4 h, 24 h, 48 h after certoparin administration. The 48 h sample was taken before application of certoparin for the next dialysis session.

In a second subgroup of 36 patients (referred to as PK-B; introduced after the amendment), additional pharmacokinetic assessments were performed after 8 weeks (visit V4). This subgroup included a target number of 6 patients for each of the following bolus doses of 3,000 IU, 3,600 IU or 4,200 IU prior to dialysis, 6 patients with a bolus of 3,000 IU plus an infusion of 600 IU/h up to 1 h before the end of the dialysis session, 6 patients with a bolus of 3,600 IU plus an infusion of 600 IU/h up to 1 h before the end of the dialysis session, and 6 patients who underwent hemodiafiltration. For PK-B samples were obtained immediately before certoparin administration (0 min), and at 2 h, the end of the dialysis session and at 48 h (at the following dialysis session respectively).

PK samples were collected from the fistula. In patients with a catheter, blood samples were taken by venipuncture. All PK samples were processed and kept frozen at -20°C or less and transferred to the central laboratory. Anti-Xa activity was measured by hydrolysis of a chromogenic peptide substrate [18] using certoparin to obtain the calibration curve [14]. The PK parameters for PK-A were determined in plasma using non-compartmental methods.

Prothrombin fragment F1/2 (Dade Behring, Germany, normal values below 230 pmol/l), thrombin-antithrombin complex (TAT, Dade Behring, Germany, normal values below 4.2 μg/l)), and D-Dimer (Technoclone, Austria, normal values below 250 ng/ml) were determined using a microplate reader (Dynatech, Germany).

### Dialyser redness

Clotting of the dialysis filter at week 8 was also assessed by a specialized central lab (eXcorLab GmbH, Obernburg, Germany). For this assessment, the filters were flushed with 1 l of saline and stored at 2-8°C before shipment to the specialized central lab. Redness of the dialysers was quantified by taking four photographs of each filter (at 0°, 90°, 180°, 270°) and analysed as the mean percentage of the clotted transversal filter (clotted : total area) by the image analysis software Image Pro plus, version 6.3 (Media Cybernetics, Inc., USA). The blue filter of the software was used to enhance redness and the flatten filter was enabled to reduce noise. The distinction between red and white areas was corrected manually using the intensity histogram and by comparing the red colour image generated on the screen with the real area of interest on the spread fibre bundle.

### Statistics

This study assessed the feasibility of certoparin as an alternative treatment option with respect to complications. Based on clinical experience, the percentage of unsatisfactory dialysis results was estimated to be in between 0 and 5% [17,19-21]. It was determined that at a sample size of 100 patients an observed 1% incidence would have a 95% CI with a width of 4% (-1 to 3%). At an observed rate of 3% the width would be 6.6% (-0.3 to 6.3%) and at 5% the width would be 8.6% (0.7 to 9.3%). This precision was regarded adequate for the purpose of this trial. An additional 10 patients were allowed to correct for the broader population (hemofiltration/hemodiafiltration) defined in the amendment. Therefore, the total sample size was 110 patients.

## Results

### Study population

**Figure 1:**
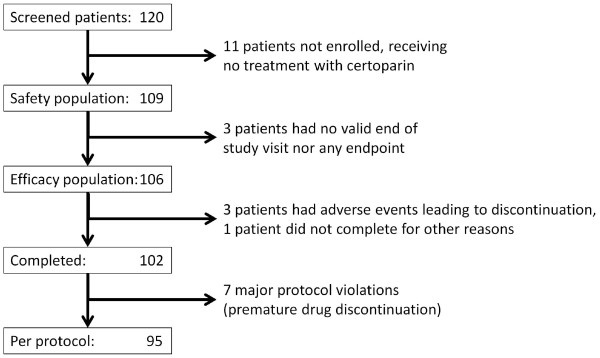
Patient flow.

A total of 120 patients were screened for their eligibility (Figure 1). Of these, 109 patients were treated with at least 1 dose of certoparin and made up the safety population. 106 patients (97.2% of 109) were available for efficacy analyses (ITT-Population). 95 patients completed the study per protocol (87.2% of 109). Table 2 displays patient characteristics of the safety population.

**Table 2:** Patient and treatment characteristics of the safety population

	**n = 109**
Age	
Mean ± SD [years]	66.3 ± 14.7
Median (range) [years]	71 (26-90)
≥ 65 years (n, %)	66 (60.6)
Male gender (%)	63 (57.8)
Weight	
Mean ± SD [kg]	82.5 ± 18.3
Median (range) [kg]	81.1 (41-151.6)
Dialysis technique	
Hemodialysis (n, %)	99 (90.8)
Hemodiafiltration (n, %)	10 (9.2)
Vascular access	
Arteriovenous fistula (%)	100 (91.7)
Permanent dialysis catheter (%)	9 (8.3)
Exposure to certoparin	
Mean ± SD [days]	61.1 ± 33.9
Median (range) [days]	57 (3-397)
Dialyses	
Mean ± SD [dialyses]	25.0 ± 4.2
Median (range) [dialyses]	25 (2-32)

SD, standard deviation.

### Dosing of certoparin

Certoparin was started at 3,000 IU and uptitrated until a satisfactory result was obtained. At the final visit 68.9% of patients received certoparin as a bolus, the majority at a dose of 3,000 IU (34.0%) and 3,600 IU (22.6%) (Table 3). Only 4 patients (3.8%) needed a dose below 3,000 IU. Body weight, haemoglobin or the prior UFH dose were not predictive of the required certoparin dose with correlation coefficients *R*^*2*^ of 0.1408, 0.0457 and 0.0068, respectively.

**Table 3:** Distribution of doses at the final dialysis (visit 4/week 8) of the ITT-Population

**Total dose (IU)**	**Patients with a single initial bolus**		**Pts with initial bolus and infusion**	
	**Bolus**	**n (%)**	**Bolus/Infusion**	**n (%)**
1200	1200	1 (0.9)		
2400	2400	3 (2.8)		
3000	3000	36 (34.0)		
3600	3600	24 (22.6)		
4200	4200	8 (7.5)		
4800			3000/1800	1 (0.9)
4950			3000/1950	1 (0.9)
5100			3000/2100	5 (4.7)
5250			3000/2250	2 (1.9)
5400			3000/2400	11 (10.4)
5550			3600/1950	1 (0.9)
5700			3600/2100	2 (1.9)
6000	6000*	1 (0.9)	3000/3000	1 (0.9)
			3600/2400	3 (2.8)
6600			4200/2400	6 (5.7)
∑		73 (68.9)		33 (31.1)

IU, International Units, ITT, intention to treat; * a bolus of 6000 IU was only allowed during PK-A.

### Efficacy endpoints

**Figure 2:**
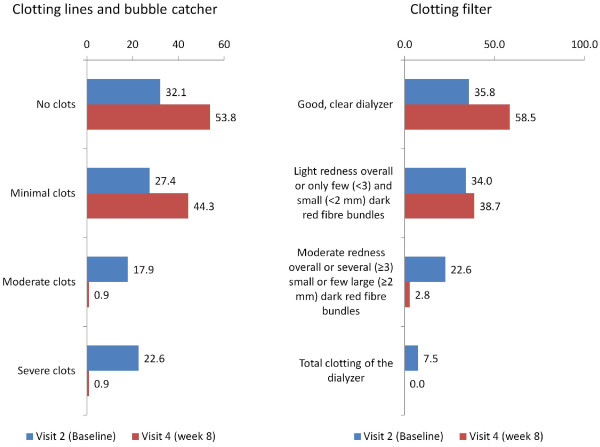
Clotting of the lines, the bubble catcher and the dialyser at baseline (V2) and after 8 weeks (V4) (results given as % of the ITT population).

**Figure 3:**
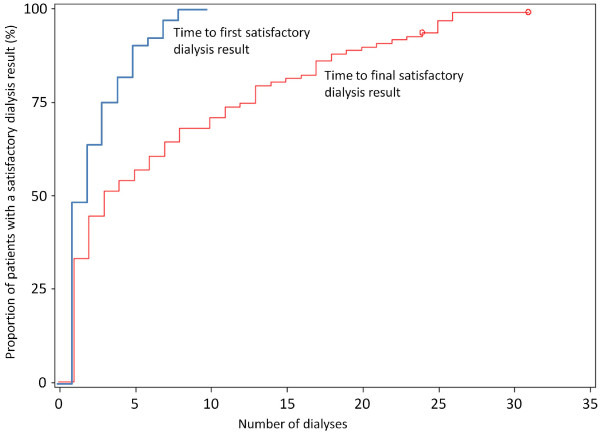
**Proportion of patients with a satisfactory dialysis result (%)**.

The primary endpoint, which was the percentage of unsatisfactory dialysis results as per investigator judgement at the 8 week visit due to clotting or bleeding, was met in 2 patients of the ITT population (2/106 patients, 1.9%; 95%CI 0.23%–6.65%) and the per protocol population (2/95 patients; 2.1%), none of these due to major bleeding. Clotting in the blood lines and bubble catcher was considered to be moderate to severe in these 2 patients. A total of 3 patients (2.8%) had moderate clotting (redness overall or several [≥ 3] small or few large [≥2 mm] red fibre bundles) of the dialysis filter at week 8 (Figure 2). A mean of 15.7 ± 14.3% of the dialysis filters’ visual surface area was showing redness at 8 weeks. The time to a first and finally satisfactory dialysis showed an exponential increase with time and demonstrates that about 50% of patients achieved a satisfactory dialysis results within the first 2–3 dialysis treatments (Figure 3).

### Antixa activity

**Figure 4:**
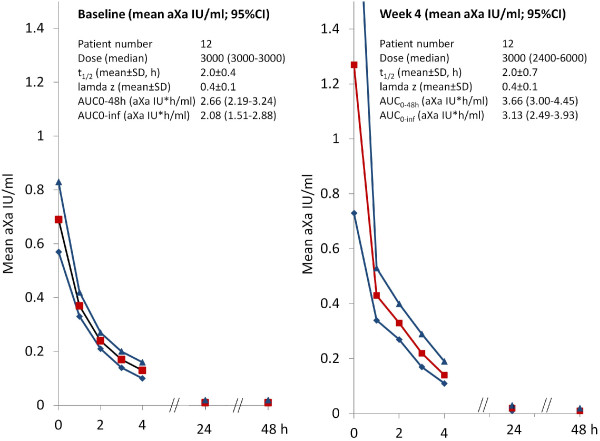
**Pharmacokinetic evaluation (PK-A population; n = 12) at baseline (V2) and 4 weeks (V3).** The first sample was obtained after 1 minute, resulting in a high peak compared to other studies in which first samples were obtained later. Legend: values at 24 and 48 h not displayed (0.01-0.02). In a number of patients these values were below the detection limit of the assay. Means are displayed in red and 95% confidence intervals in blue.

In the PK-A subgroup (12 patients), aXa activity was determined at baseline and at the 4 week follow-up (Figure 4). Peak activity was 0.69 (95%CI 0.57–0.83) aXa IU/ml at minute 1. At 24 and 48 h, aXa activity was low (0.01–0.02) with a number of patients below the detection limit. Logarithmic transformation resulted in an almost linear kinetic (except for the activity determined at 1 min; plot not shown).

**Figure 5:**
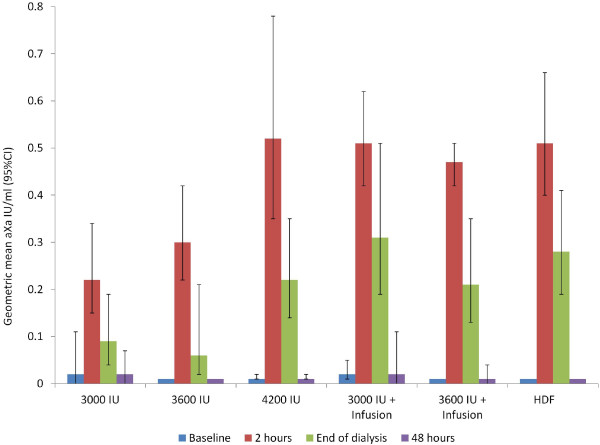
**Pharmacokinetic evaluation (PK-B population; n = 36) at week 8 (V4).** Legend: HDF, haemodiafiltration; HDF patients received median dose of 5400 IU.

In the PK-B subgroup (36 patients different from PK-A), aXa activity was monitored at the 8 week visit and results grouped by certoparin dose and dialysis technique used (hemodiafiltration) (Figure 5). The results demonstrate larger confidence intervals (vs. the PK-A group), no sign of certoparin accumulation, a low dose dependency and comparable levels with hemodiafiltration.

**Figure 6:**
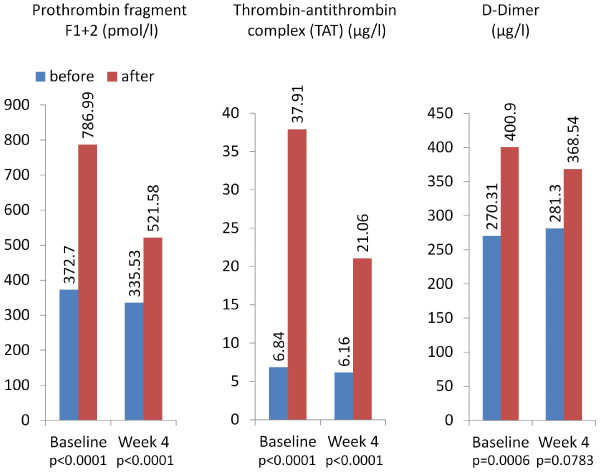
Coagulation markers (Safety population*). Legend: *109 patients were available for evaluation at baseline (V2) and 103 patients at week 8 (V4) .

Results in the PK-A and PK-B subgroups were rather consistent despite differences in centres involved and the time frame monitored (8 vs. 4 weeks). At baseline (n = 12; PK-A; median dose 3000 IU, range ±0), at week 4 (n = 12, PK-A; median dose 3000 IU, range 2400–6000) and at week 8 (n = 36; PK-B; median dose 3000 IU, range 2400–6600), median plasma aXa levels 2 h after administration were 0.24, 0.33 and 0.38 aXa IU/ml. C_48h_ was 0.01 aXa IU at all visits (below detection limit in 50%, 66.7% and 82.4% of patients).

### Coagulation markers

Prothrombin fragment F1 + 2, thrombin-antithrombin complex (TAT) and D-Dimer (Figure 6) showed that the activation of coagulation was also present with the use of certoparin, although the increase between pre- and post-dialysis in most of these parameters was almost halved at week 4.

### Bleeding complications and adverse events

In 3.0% during 2724 dialyses, prolonged fistula compression times were reported which corresponded to 45 patients and 83 cases. Eight patients (7.34%) had at least one episode of minor bleeding (total cases 11) but none experienced major bleeding complications. These were mouth, gastrointestinal and rectal hemorrhage (1 event each), epistaxis (3 events), hematoma (2 events), arteriovenous fistula site haematoma (2 events) and arteriovenous fistula site hemorrhage (1 event).

There were a total number of 270 adverse events (AE) in 93 out of 109 patients (85.3%); 32 were suspected to be drug related. Of 10 adverse events which were considered to be serious, none was suspected to be related to the use of certoparin: one of these patients with a previous cardiac arrest (after 3 study weeks) died. Other SAEs were renal transplantation (3 patients, considered because of hospitalization), angina pectoris, atrial fibrillation, atrial flutter, gastrointestinal haemorrhage, chest pain, fracture, injury, hypoglycaemia, necrosis of an extremity and peripheral arterial occlusive disease.

## Discussion

The low molecular weight heparin certoparin appears to be effective and safe for anticoagulation of patients on maintenance hemodialysis based on the results of this multicenter, open-label and prospective but non-controlled trial. The following findings are noteworthy: 1) a satisfactory dialysis result was achieved in 98% of patients with no bleeding complications requiring premature interruption of the hemodialysis session or major bleeding; 2) the majority of patients needed a bolus dose of 3,000 or 3,600 IU certoparin only which resulted in a satisfactory dialysis result within 2-3 sessions suggesting a simple dosing regimen; 3) there was no need for adjusting the certoparin dose based on body weight; 4) there was no sign of certoparin accumulation even at higher doses; 5) in only 3% of dialyses, prolonged fistula compression times were observed.

### Efficacy and safety

From a clinical perspective, a successful dialysis session is characterized by the absence of clotting or major bleeding as well as stable dosing during long-term use. However, there is no established margin for an acceptable rate of complications against which the rate observed in this study could be compared. Previous work published by Schrader et al. reported thrombosis rates of the extracorporeal circuit of 1.33% for UFH and 1.59% for dalteparin [19]. Saltissi et al. observed that 3.06% of dialyses with UFH and 1.53% of dialyses with enoxaparin showed clotting (5 or more on a 10 point scale), severe bleeding events were infrequent with incidence rates of about 0 or 0.1% [20]. Bramham et al. reported thrombosed circuits in 2.2% of UFH and 0.7% of tinzaparin treated patients with major bleeding events between 0 and 0.26% [21]. Finally, the meta-analysis published by Lim et al. revealed that about 10-11% of patients had any bleeding complications during dialysis [17]. The only case of major bleeding they reported was a patient that had access site bleeding after hemodialysis with tinzaparin; however, the patient’s activated partial thromboplastin time was elevated and the investigators concluded that the patient accidentally received additional UFH [21]. The incidence of circuit thromboses in this meta-analysis was 2.2% with LMWH and 1.9% with UFH [17]. Against this background, it appeared reasonable to aim at determining the proportion of patients with complications during dialysis defined as the presence of clotting or bleeding using a straightforward, non-controlled design. With an incidence of 1.9% (ITT, 2.1% PP) and no major bleeding reported and a confidence interval of 0.23-6.65%, we were thus able to confirm previously reported rates for other low molecular weight heparins [19,20,22].

Another matter of practical concern is prolonged fistula compression times with the use of heparins. Most prior studies reporting this variable documented between 5 and 13 minutes of fistula compression to be necessary with either LMWH or UFH [17]. Differences between both treatment options were either negligible or in favour of the LMWH (shorter compression) [23-25]. In our study, 45 patients (41.3%) had prolonged fistula compression times with a total of 83 cases (3.0% during 2724 dialyses). This appears reasonably low from a clinical perspective, but, unfortunately, the degree of “prolongation” was not predefined because of the high clinical variability and left up to the treating physician potentially resulting in overestimation of this endpoint.

### Dosing of certoparin

The majority of patients in our study needed a bolus dose of 3,000 or 3,600 IU certoparin, which resulted in a satisfactory dialysis result within 2-3 sessions. Overall 68.9% of patients received a bolus up to 4,200 IU only and the remaining patients a combination of bolus/infusion. Only 4 patients needed an even lower dose than 3000 IU. This dose reduction was however not enforced by the study protocol. This is in principal agreement with the experience made with other LMWHs, for which a single bolus administration without any heparin priming is usually sufficient for effective dialysis [21,26,27]. Davenport reported that, due to its long half-life, a bolus dose of 0.8 mg/kg enoxaparin is usually sufficient for about 98% of patients [2]. Tinzaparin, which has a shorter half-life than enoxaparin, requires two injections in more patients when dialysing more than 4 but less than 6 h and in the majority of patients dialysing for more than 6 h [2]. In his most recent review Davenport reports on the possibility to reduce enoxaparin to 0.4 mg/kg bolus and reduce tinzaparin to single bolus of 1500 IU to be sufficient as anticoagulation for an intermittent hemodialysis, especially in patients with the risk constellation for bleeding [3].

The standard dose for the initiation of certoparin was 3,000 IU which was uptitrated in case of relevant coagulation. At uptitrated doses of certoparin, the coagulation system was found to be still activated, although the increase in prothrombin fragment F1 + 2, thrombin-antithrombin complex (TAT) and D-Dimer was almost halved at week 4. These numbers are well comparable with data published by Milburn et al. who measured TAT and D-Dimer in 55 patients undergoing hemodialysis receiving UFH [28]. The elevated TAT values 31.07 μg/ml during dialysis at baseline were only half as high at visit 4 (14.90 μg/ml). The differences for D-Dimer were similar (130.59 vs. 87.23 μg/l). In a study by Sagedal et al. using dalteparin for hemodialysis in 12 patients, a number of coagulation parameters were determined [29]. TAT, prothrombin fragment F1 + 2, antithrombin (coagulation), plasmin-antiplasmin complex (fibrinolysis) and β-thromboglobulin (platelet activation) were elevated at baseline to the same extent as in our study at baseline. However, no repeated measurements during long-term dialysis were presented.

### Anti-xa activity

In routine clinical practice, anti-Xa activitiy is not usually monitored because of practical limitations, but clinical inspection of the dialyser and bubble trap is used instead to adjust dose. However, its determination allows assessing the degree of anticoagulation and determining the pharmacodynamics of anti-Xa activity. In our analyses, we documented a high initial anti-Xa activity at minute 1 after the injection which sharply declined thereafter with activities of about 0.4 IU/ml at 1 h and < 0.2 IU/ml at 2 hours. This latter concentration is comparable with current clinical practice recommendations that use targets between 0.2-0.4 IU/ml, particularly in patients with increased risk for bleeding [30]. However, this concentration is lower than recommendations for an anti-Xa activitiy for the initial treatment of thrombosis (0.4-0.6 IU/ml) [31]. There was no evidence of an accumulation in our study even at high doses or with additional certoparin infusion, which fits with previous data for UFH, dalteparin, tinzaparin and enoxaparin [19,32]. The data are consistent with prior data on certoparin in therapeutic doses (8.000 IE bid s.c.) [14]. The area under the curve was 5.588 IU*h/ml which corresponds to 2.096 IU*h/ml referring to the lower dose of 3,000 IU in the present study.

Compared to other LMWHs, certoparin appears to have a rather fast decline of anti-Xa activity, which is good from a safety perspective. Guillet et al. reported for enoxaparin that a single injection of 60 IU/kg led to an anti-Xa activity higher than 1.2 IU/ml during the first 2 h, and between 0.4 and 1.2 IU during the hours 3 and 4 [20]. After the end of dialysis, anti-Xa activity remained high 10 hrs (0.4 IU/ml) and 24 hrs (0.1 IU/ml) after injection. This may suggest an increased risk of bleeding for a prolonged time period after cessation of dialysis. In a comparative pharmacokinetic study of dalteparin 2,500 IU, enoxaparin 40 U/kg and danaparoide 34 U/kg mean aXa activities were reported 4 hrs post injection with 0.2 IU/ml for dalteparin, 0.38 IU/ml for enoxaparin and 0.54 IU/ml for danaparoide, respectively [32]. On the other hand the fast elimination of certoparin may explain the more frequent need for an infusion in addition to the initial bolus (31.1%) as opposed to enoxaparin for example for which a repeated bolus is necessary in only about 2% of cases [2].

### Limitations

The MEMBRANE study results are limited by the following considerations: 1) The MEMBRANE study design was not controlled with placebo, UFH or any other LMWH. This may be perceived as a limitation but appeared justified based on the existing data with other heparins [17] and the low incidence rate of complications expected. 2) The study included patients with different residual renal function which may have interfered with certoparin half-life. 3) The degree of “fistula compression time prolongation” was not predefined. This was because of the potentially high clinical variability which was and left up to subjective assessment of the treating physician.

## Conclusions

Certoparin appears to be effective and safe for anticoagulation in patients undergoing maintenance hemodialysis. Bleeding complications were rare and prolonged fistula compression necessary in only 3% of dialyses. With a simple titration scheme with about two thirds of patients needing a single bolus, certoparin is a valuable alternative to other existing treatment options whose dosing regimen is more complicated or requires complex dose adjustment.

## Competing interests

Oliver Dorsch, Detlef H. Krieter, Horst-Dieter Lemke, Peter Bramlage, Stefan Fischer and Job Harenberg disclose to have received research support or honoraria for advisory board and lectures from a number of pharmaceutical companies producing low-molecular-weight heparin including Novartis. Nima Melzer and Christian Sieder are employees of Novartis Pharma GmbH, Nürnberg, Germany.

## Authors’ contributions

OD, DHK, HDL, NM, CS, SF and JH have been involved in the conception and design of the study. CS was responsible for the analysis of data in cooperation with PB. PB and OD have drafted the manuscript and all other authors have been revising the article for important intellectual content. All authors have finally approved the version to be published.

## Pre-publication history

The pre-publication history for this paper can be accessed here:

http://www.biomedcentral.com/1471-2369/13/50/prepub
